# AI in Breast Cancer Imaging: A Survey of Different Applications

**DOI:** 10.3390/jimaging8090228

**Published:** 2022-08-26

**Authors:** João Mendes, José Domingues, Helena Aidos, Nuno Garcia, Nuno Matela

**Affiliations:** 1 Faculdade de Ciências, Instituto de Biofísica e Engenharia Biomédica, Universidade de Lisboa, 1749-016 Lisboa, Portugal; 2 Faculdade de Ciências, LASIGE, Universidade de Lisboa, 1749-016 Lisboa, Portugal

**Keywords:** breast cancer, machine learning, deep learning, self-supervised learning, data augmentation, automatic detection, risk prediction

## Abstract

Breast cancer was the most diagnosed cancer in 2020. Several thousand women continue to die from this disease. A better and earlier diagnosis may be of great importance to improving prognosis, and that is where Artificial Intelligence (AI) could play a major role. This paper surveys different applications of AI in Breast Imaging. First, traditional Machine Learning and Deep Learning methods that can detect the presence of a lesion and classify it into benign/malignant—which could be important to diminish reading time and improve accuracy—are analyzed. Following that, researches in the field of breast cancer risk prediction using mammograms—which may be able to allow screening programs customization both on periodicity and modality—are reviewed. The subsequent section analyzes different applications of augmentation techniques that allow to surpass the lack of labeled data. Finally, still concerning the absence of big datasets with labeled data, the last section studies Self-Supervised learning, where AI models are able to learn a representation of the input by themselves. This review gives a general view of what AI can give in the field of Breast Imaging, discussing not only its potential but also the challenges that still have to be overcome.

## 1. Introduction

### 1.1. Breast Cancer: Statistics and Risk Factors

Breast cancer(BC) surpassed lung cancer as the most commonly diagnosed cancer, with approximately three million cases diagnosed in 2020 and nearly seven hundred thousand deaths [1]. The incidence of BC has been increasing yearly since the mid-2000s [2]. Nonetheless, in terms of mortality, a decreasing trend has been observed in recent years. However, this trend did not affect every ethnicity in the same fashion. For example, while the incidence of BC remained higher for Non-Hispanic White (NHW) people than for Non-Hispanic Black (NHB) people across the years, the death rate decreased much more for NHW than for NHB. There is such a great difference in these decreases that while the incidence remains higher for NHW, the death rates are higher for NHB [3].

Although age is the most studied risk factor for the development of BC—so much so that screening programs are based upon it—there are several elements that contribute to the emergence of this disease. High Body Mass Index, prior history of neoplastic/hyperplastic breast disease, and the existence of BC family history are important risk factors [4]. Prolonged lifetime exposure to estrogen—early menarche and/or late menopause—is also a risk factor to consider [5]. Given that, the use of oral contraceptives can also increase the risk of developing BC [6]. In addition to that, genetic mutations such as in the BRCA1/2 gene put women at higher risk of developing this disease. Among several texture characteristics that are related to risk, percent of mammographic density (%PMD) presents itself as one of the most studied. Actually, women with dense breasts (60–70%PMD) are at four to five times higher risk than women with fatty breasts [7,8,9].

### 1.2. Screening and Commonly Found Lesions

Mammography is the most used imaging technique in screening programs. This imaging technique helped to detect life-threatening cancers earlier, improving prognosis, and decreasing mortality rates up to 50%. Consequently, the fact that cancer is detected earlier leads to less aggressive treatment and fewer money losses from the patients [10,11]. Despite its positive impacts, mammography has some flaws. For example, in the United States of America, all screened women will experience at least one false positive in their life. Actually, an overview of the benefits and harms of mammography showed that in 1000 women that have biennial mammography, 200 of them will have a false positive, 15 will be overdiagnosed—meaning that cancer that could not harm the women during their lifetime will be diagnosed—and 3 will have interval cancers—a lesion developed between sequential screenings. While a false positive negatively impacts women’s mental health, resulting in unnecessary anxiety, interval cancers have a direct influence on physical health. Diagnosis timing is of extreme importance when it comes to cancer, so it is important to understand if the interval cancer was in fact developed between screenings, or if it was missed during the last screening assessment due to mammography flaws [12]. The fact that mammography is a 2D image leads to tissue overlap, which can result in both lesion masking, or fake lesion creation—hence resulting in false positive and false negative results. In addition to that, mammography is known to diminish its sensitivity for dense breasts (30–64%) in comparison with fatty breasts (76–98%)—which is a problem, since it is established that women with dense breasts are at higher risk of developing BC [10].

The use of other imaging techniques along with mammography can help to overcome some of its problems. Ultrasound combined with mammography can increase cancer detection, with the upside that ultrasound works slightly better for dense breasts [10,13]. Tomosynthesis, which is acquired with a moving X-ray machine, allows overcoming the problem of overlapping tissue. In addition to that, when combining tomosynthesis and mammography, cancer detection can increase by 30–35%, with the advantage that tomosynthesis is better in lesion characterization and tumor staging [10]. Magnetic Resonance Imaging (MRI) can be used instead of mammography for women with a higher risk of developing the disease and has higher sensitivity as it is not affected by breast density [14]. Limited capacity to find certain types of lesions, higher acquisition and reading time, and higher costs are some of the limitations of ultrasound, tomosynthesis, and magnetic resonance, respectively [10,13,15].

There are two main findings in terms of breast lesions, especially in what concerns computer vision applications: masses and microcalcifications. Masses are defined as space-occupying lesions and are assessed with regard to their shape, margins, and density. Concerning shape, masses can be round, oval, or irregular, with the probability of malignancy increasing as the shape becomes irregular. The margins can be classified as circumscribed, obscured, microlobulated, indistinct, or spiculated, with the latter representing the highest probability of malignancy. In terms of density, the higher it is, the higher the likelihood of malignancy. Microcalcifications, on the other hand, are small deposits of calcium that are assessed in terms of distribution on the breast and morphology. The morphology can be: round and punctuate, which is typically benign; amorphous or coarsely heterogeneous, which is an intermediate state between being benign and malignant; or fine pleomorphic/fine linear, which indicates a higher probability of malignancy. As for distribution, a higher probability of malignancy occurs when the calcifications are arranged in lines [16,17].

### 1.3. The Role of Artificial Intelligence in Medical Imaging

Artificial Intelligence (AI) can play a major role in improving image interpretation and diagnostic outcomes [18]. There are two sub-fields of AI that need to be addressed in order to fully grasp medical applications: Machine Learning and Deep Learning.

Machine Learning (ML) was defined as the field that gives computers the ability to learn without being expressly programmed. Hence, ML can build models from input data and make data-oriented predictions. In addition to making predictions, ML can be used to find important structural information in data and unveil hidden patterns.

Deep Learning (DL) is itself a sub-field of ML that is focused on data representation in the most optimized fashion possible in order to simplify the learning task. One of the main differences between DL and ML is that while DL can learn using raw data as input, in ML the learning process needs handcrafted features that represent the input data to learn. Thus, learning in DL is more automatic [19,20].

The potential of AI in the medical imaging field covers several applications. For example, imaging systems could be improved with AI systems, not only optimizing acquisition timing but also improving position and helping to characterize the findings. At the same time, automatic detection of lesions has been studied in different medical fields such as breast conditions, pulmonary and thyroid nodules, and prostate cancer. Furthermore, these AI detection systems have been tested with several different imaging techniques such as ultrasound, MRI, and tomosynthesis. In addition to automatic detection, lesion interpretation can also be performed with AI systems. Computer-Aided Diagnosis (CAD) systems are able to serve as a second opinion to radiologists/image interpreters, improving the diagnosis and prognosis of the found lesions. This interpretation is usually in normal/abnormal cases but can be extended to more complex functions, saving precious time for image analysts while improving their performance. Finally, AI has its role in image post-processing and quality analysis such as, for instance, in image registration and volume segmentation in several imaging modalities [18,21].

### 1.4. Related Work

In order to produce the scoping review presented in this paper, a background literature review was performed. The presented review is innovative as it presents a generalized overview of various applications of AI in breast imaging. In addition to the common approaches of lesion detection in mammograms, works in alternative imaging modalities are explored. Research in the field of risk prediction—in line with the current medicine paradigm—is also included in the paper. In addition to that, data augmentation approaches that help to overcome some of the usual obstacles in the development of AI solutions with medical images were also reviewed. Finally, the innovatory use of self-supervised learning was deeply reviewed as well. Since this paper aims to review different applications of AI in breast imaging, it was decided that, instead of having extensive subsections, articles that gave a general view of the methodologies usually used should be reviewed. For Section 2.3.1, concerning the works that used mammograms, the goal was to give a general view of how AI can be applied to lesion classification. Hence, it was our target to show not only the use of different algorithms and evaluation metrics but also different dataset construction. Given that, a search in Google Scholar with the terms “texture”, “mammogram”, and “classification” led to the screening of several articles that were screened based on their good readability, methodology, results, and data used. The inclusion of the keyword “support vector machine” was inputted later in order to include studies that used this approach. Considering these criteria, two studies were included since they present a general view of Machine Learning applications in lesion classification through well-known datasets (DDSM and MIAS), while using different methods for dataset construction, algorithm application, and model evaluation. For Section 2.3.2, the goal was to show that similar methodologies can be applied to different imaging modalities. Therefore, the use of the keywords “breast tumor”, “ultrasound”, “texture”, “classification”, and “automatic detection” were used for ultrasound. For MRI, the keywords were “breast mri”, “lesion classification”, and “texture”. Among the several articles that came out, they were screened in order to verify if the methodologies applied were similar to what was conducted in Section 2.3.1. The reviewed papers were the ones that matched the said methodologies. For Section 2.3.3, different combinations of the following keywords were used: “breast cancer risk”, “mammography”, “machine/deep learning”, “parenchyma/texture”, and “patterns/features”. The articles were included with the rationale of showing the different methodologies (differentiating risk groups, directly assessing risk in a mammogram, using a single ROI vs. the entire mammogram, etc.) that both Machine Learning and Deep Learning can have in the field of risk prediction.

The article search process for the “AI and Data Augmentation” section worked similarly to a “FOAF” (friend-of-a-friend) search, where in most cases multiple articles that were referenced from the current analyzed article were analyzed. The Google Scholar tool was used to search for key terms such as “Generative Adversarial Networks Medical Imaging”, “Generative Adversarial Networks Breast Cancer”, “Image Generation Medical Imaging”, “Image Generation Breast Cancer”, “Single Image Generation Medical Imaging”, and “Single Image Generation Breast Cancer”. For each search term, the results were ordered by date and relevance and analyzed based on the work abstract. If the abstract was convincing, the team would perform a more in-depth analysis of the paper, otherwise, the team would skip it. Each analyzed paper, as well as the papers referenced by them, were added considering various aspects that were defined to mark a paper as more adequate to the study. These aspects were: the ease of reading of the paper as well as understanding how the project worked; the definition of a dataset and how they acquired it and pre-processed it; a good definition of a methodology of work; if it made significant improvements to some other base work or developed something completely new; a robust definition of the project implementation; well-presented metrics and good results for those metrics; the presence of limitations and future improvements; and amount of impact that the work made for the presented specific problem. Similarly, for Section 4 on Self-Supervised Learning, the search included the terms “Self supervised learning” combined with “breast cancer” and “mammography“. We identified the most recent papers, published in known venues, and used the platform feature that shows similar papers to these in order to identify other possible candidates.

The organization of the remainder of this paper is as follows: Section 2 describes AI general methods in the interpretation of lesions in different imaging modalities. Section 2.1 and Section 2.2 analyze commonly used features in Machine Learning research. Section 2.3 reviews works in lesion interpretation—usually categorization of images into benign or malignant categories—for different imaging modalities and extends this review to tissue analysis in the field of breast cancer risk prediction. Section 3 is concerned with different work related to augmenting data availability, while Section 4 with recent advances in the field of Self-Supervised Learning. Section 4 and Section 5 correspond to a discussion of the presented results and a conclusion about the work conducted, respectively.

## 2. Traditional AI in Lesion Detection and Tissue Interpretation

Lesion detection and interpretation are often found together: from studies that differentiate healthy tissue from cancer lesions to investigations that aim to distinguish benign from malignant lesions, there are several fields where AI plays a part. Since mammography is the most commonly used imaging technique, it is expected that most of the research is focused on it.

### 2.1. Texture Features

As it was already discussed, handcrafted features are important for ML applications, and the field of lesion interpretation is no different. Most of the works use different texture features that represent the variation among pixel intensities, denoting the architecture of the analyzed object [22]. The relationship between breast tissue texture and breast cancer was first described by Wolfe [23], where he defined four different breast texture patterns:

- N1—Lowest Risk, parenchyma is mainly composed by fatty tissue without visible ducts.
- P1—Low Risk, ducts may occupy as far as a quadrant of the breast.
- P2—High Risk, there is a “severe involvement” of ducts that occupy more than 25% of the breast.
- DY—Highest Risk, the severe involvement seen in P2 is accompanied by dysplasia.

The categorization of a breast to each specific category is not objective once it depends on the interpretation of the healthcare professional that is doing the assessment [24]. In addition to the studies provided by Wolfe, other researchers [25] have also linked parenchyma texture pattern features with the risk of developing breast cancer. Because of the fact that there are relationships between fatty and glandular tissue that are described by texture and that can be associated either with a healthy or a disease condition, several texture descriptors were studied and developed. These descriptors can be divided into different subgroups that are described further on.

#### 2.1.1. Co-Occurrence Features

**Figure 1:**
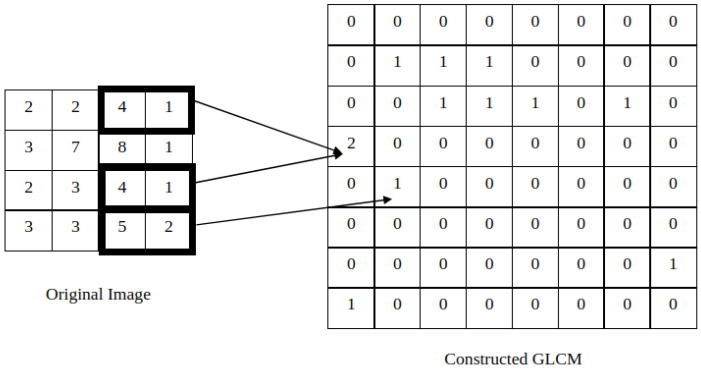
Construction of the GLCM for the 0° direction.

Co-occurrence features were first described by Haralick, Shanmugam, and Dinstein with the goal of classifying pictorial data. The idea was to create features that could describe the relationship between neighbor pixels and, from that, the concept of the gray-level co-occurrence matrix (GLCM) was developed. Given an image with N gray levels, the corresponding GLCM will have size (N,N). Then, the entry (i,j) of the GLCM represents how many times a pixel of intensity i appears near a pixel of intensity j in the original image, in a given direction. For instance, in Figure 1, it is possible to verify that, in the original image, the pair (4,1) occurs twice in the 0 direction and, for that reason, the entry (4,1) of the GLCM is 2. For the same reason, the entry (5,2) of the GLCM has the value 1. The same rationale can be applied to other directions, creating different GLCMs. It is the mathematical manipulation of the GLCM—through specific formulas described in Haralick’s research—that allows the extraction of several texture features [26,27].

#### 2.1.2. Run-Length Features

**Figure 2:**
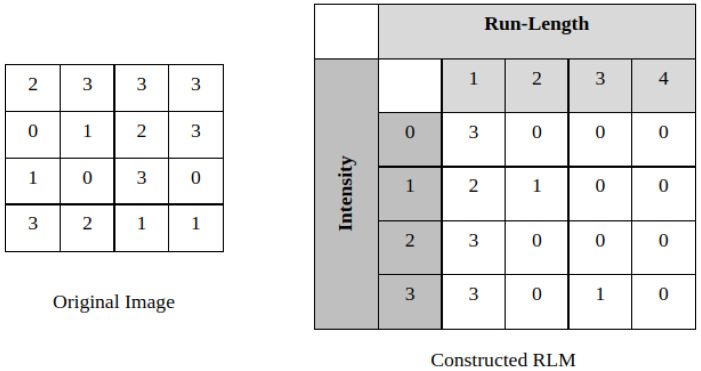
Construction of the RLM for the 0° direction.

Opposite to what happens in co-occurrence features, here, pixel pairing is not used for feature calculation but rather sequences of pixels with the same intensity, hence, run length. Once again, feature extraction is dependent on a previous matrix construction, in this case, a Run-Length Matrix (RLM). Here, the entry (i,j) of the RLM will represent the number of times that a run length of size j for the pixel intensity i occurs. For example, when analyzing Figure 2, it is possible to see that, in the 0 direction, the intensity 3 appears alone three times. Therefore, the entry (3,1) of the RLM has the value 3. On the other hand, intensity 3 appears one time in a sequence of three pixels. For that reason, the entry (3,3) of the RLM has 1 as its value. As for the GLCM, not only the same rationale can be applied for other directions, as it is the manipulation of the RLM that allows feature extraction [28].

### 2.2. Additional Features

The previously addressed groups are the two most widely used in ML studies concerning BC detection, however, there are other groups of texture features that can be used. Characteristics computed through the power spectrum such as its First Moment or its Root Mean Square are described in the literature as feature descriptors [29]. The relationship between a pixel and its neighborhood can be well-described through a Local Binary Pattern (LBP), which explains why LBP is used in texture analysis. This is how the definition of an LBP works: Given a specific neighborhood, the region around the central pixel will be analyzed in a pixel-by-pixel basis. If a pixel has a higher value than the central pixel, then it will be assigned the value 1, otherwise it will receive 0 as its value. After that processing is completed, the resulting pixel values will be concatenated in an anti-clockwise direction into one binary identifier that can afterward be converted to a decimal [30].

Morphological features are often used to assess tumor characteristics, as they occur in a work proposed by Chen et al. [31]. Benign tumors often have a different morphology in comparison with malignant tumors—especially, the borders of the lesion tend to be smoother and more regular. Therefore, the authors develop and explain several features: area—typically malignant tumors have larger areas; circularity—the closer this value is to one, the more regular the shape of the tumor; compactness—looks for an overlap ratio between the tumor area and a circle positioned at the center of the tumor; eccentricity, the common mathematical metric and some variations of this metric (elliptic-normalized circumference; and elliptic–area ratio); and some metrics whose names are self explanatory (and deeply described in the paper): roundness; number of substantial protuberances and depressions; lobulation index; and aspect ratio.

### 2.3. AI in Breast Imaging Analysis

#### 2.3.1. Machine Learning in Mammography Lesion Interpretation

Most works in this area of lesion classification/interpretation aim for a benign/malignant classification. Given that, a group of researchers [32] aimed to classify mammograms into three groups: normal, benign, and malignant. In order to achieve that, they used a database which is composed of 322 mammograms (126 normal, 60 benign, and 48 malignant). For the test set, 37 normal, 23 benign, and 18 malignant mammograms were used, while the remaining composed the training set. In order to improve image quality, the authors used a technique called Contrast-Limited Adaptive Histogram Equalization. For simplicity of analysis, a Region of Interest (ROI) was defined and separated from the original image. In order to characterize the mammograms, GLCM texture features were extracted. It should be noted that different GLCM were constructed in four directions (0°, 45°, 90°, and 135°) and using two neighborhood distances 1 and 2. Once descriptors were extracted, the authors aimed to classify the mammograms. Since the available software only allowed a binary classification, they aimed for a two-way classifier, which was chosen to be a Support Vector Machine (SVM). So, what these two-way classifiers mean is that the SVM was trained twice: once for differentiating between normal and abnormal tissue and another time for differentiating between benign and malignant tissues among the images that were classified as abnormal. In order to diminish the dimensionality of the problem, the authors computed the average of each feature across different directions and distances. This process resulted in fifteen different features. When wanting to evaluate a mammogram, the image is given to the classifier and it is classified as normal or abnormal. If it is normal the system stops, however, if it is classified as abnormal, then the image being analyzed will be fed to the second SVM in order to be allocated into one of the remaining two classes: benign or malignant. They tested the SVM for both the first and the second stages of classification. While for the differentiation between normal/abnormal tissue the classifier achieved an accuracy of 100%; the sensitivity and specificity of the malignant/benign classification were 94.4% and 91.3%, respectively. Despite its positive results, there are some limitations concerning this research: in addition to the limited size of the test set used to evaluate performance, the fact that the test set comes from the same distribution as the training does not allow to understand how good the generalization capacity of the developed model is.

The differentiation between malignant and benign lesions was also the aim of the work of Mohanty et al. [33]. The authors used a dataset from the University of Florida, which consists of digitized mammograms with the positions of the masses annotated by an expert radiologist. These annotations served as guides for ROI extraction. In order to improve image quality, a low-pass filter that preserved important structures while suppressing irrelevant information was applied. Here, while the training set is composed of normal and cancer images (88 of each), the testing set is composed of malignant and benign images (23 and 55, respectively). Nineteen features were extracted from the previously defined ROI and were divided between two groups: GLCM and RLM. After feature extraction, the Decision Tree algorithm was used for ML model construction. In order to reduce the possibility of overfitting and to increase the model generalization capacity, boosting, winnowing, and pruning were used. Their results in classifying benign and malignant lesions using 8 GLCM features and 11 RLM features were very positive, with accuracy achieving a value up to 96.7%. In addition to that, the Area Under the Curve (AUC) retrieved from the Receiver Operating Characteristic (ROC) curve was 0.995. These results show that not only is the use of texture features important for lesion interpretation, but the use of a Decision Tree allows the development of systems that can correctly classify mammograms based on explainable features. On the downside, in order to apply this approach, laborious ROI definition and pre-processing techniques must be applied, which is a problem when translating these solutions into clinical practice: although providing positive results, it increases the workload of healthcare professionals that are already burdened with loads of work.

#### 2.3.2. Lesion Interpretation with Alternative Imaging Modalities

Ultrasound has also made its way into AI applications. A group of researchers also aimed to differentiate malignant from benign tumors in ultrasound images, using ML [34]. They used 1061 images, where 589 had malignant lesions and 472 presented benign tumors. The first step of their work consisted of ROI definition, which was performed manually when images came from different machinery. Since one of the characteristics of this imaging modality is low contrast, imaging enhancement was aimed before feature extraction. In this work, in addition to texture features, morphological features were also extracted. Compactness, which takes into account the area and the perimeter of the lesions, is a measure that can describe how smooth or complex a structure is—which is an important attribute to quantify when assessing malignancy. Radial Range Spectrum, which is able to assess the edges of a lesion, can also be useful when differentiating malignant (rough edges) and benign (smooth boundary) lesions. In terms of texture features, GLCM (0, 45, 90, and 135 degrees) and LBP descriptors were extracted along with histogram measures. The images that constituted the dataset were divided into training and testing, features were extracted and then normalized. The features retrieved from the training set were used to train an SVM classifier. After that, the trained classifier aimed to classify the instances present in the testing set. Several metrics were used to assess classifier’s performance when using both morphological and texture features: sensitivity (87.04%); specificity (87.62%); precision (87.85%); accuracy (87.32%); and AUC (0.9342). The positive results provided by this paper not only allow to understand that morphological features might be useful when trying to differentiate different types of lesions but also unveil the good capability that AI has when used with images that have lower quality. Once again, the translation into clinical practice is difficult because ultrasound is not a standard imaging technique for breast cancer diagnosis. In addition to that, the need for an ROI definition might also compromise the use of these solutions in healthcare centers. Nonetheless, it should be interesting to verify if the obtained results stand when using ultrasounds from different distributions and from women of diverse ethnicities.

Magnetic Resonance Imaging (MRI) can also play a role in the field of AI medical solutions. Just as an example, a group of researchers [35] aimed to use both morphology and texture features to correctly diagnose lesions—between malignant and benign—in breast MRI. The authors used 28 images with benign lesions and 43 images with malignant lesions. Lesion segmentation was performed first by an operator that defined an ROI where the lesion was located, then, lesions were enhanced using a filtering routine. Finally, fuzzy c-means was used to outline the lesions that were present inside each defined ROI. Morphology features, such as the previously explained compactness and texture features (GLCM), were extracted from the pre-processed images. In total, eight morphological and ten GLCM features were extracted. The chosen classifier was an artificial neural network, which was also used for choosing the best set of parameters among the extracted features. The artificial neural network architecture had one input layer with three nodes, one hidden layer with two nodes, and one output node that ranged from 0 to 1. A value of 0 means “absolutely benign”, while a value of 1 means “absolutely malignant”. The performance of the classifier was evaluated using ROC analysis. The authors trained two different classifiers: one using the entire dataset and another using only half of the dataset (14 benign and 22 malignant) and compared them in terms of performance. For the first one, the performance was evaluated through leave-one-out cross-validation, while for the second, the other half of the dataset was used for validation. When trying to develop the classifier, using the entire dataset, only with morphological features (8), three of them were chosen as relevant, and the achieved AUC was 0.8. When doing the same but only for the texture features (10), three of them were chosen as relevant, and the obtained AUC was 0.78. When combining the three selected features from each feature class, the AUC increased to 0.86. Then, when training the classifier with only half of the dataset and considering all the extracted features, five were chosen (2 morphological and 3 GLCM). An AUC of 0.93 was achieved with the training set. When considering the other half that was not used for training, the AUC decreased to 0.82. It is important to note that the 5 selected features, when using half of the dataset, were among the 6 selected features in the previous approach. This fact, allied to the good results that were obtained, shows not only the impact that AI can have in diagnosis with MRI but also the robustness of this approach. Nonetheless, the authors point out some limitations of this study: malignancy is usually related to speculation, which should be assessed with different groups of features (Fourier analysis, for instance); while here the lesions were assessed slice by slice and then averaged, other researchers reconstruct the volume and make a 3D analysis, which might be more accurate.

While the analyzed research presents several limitations—limited dataset size; test and training sets coming from the same distribution; laborious ROI definition; or the use of modalities that are not widely used in screening—they serve as proof of concept of how AI can impact the diagnosis of BC across different modalities. The incorporation of these solutions in clinical practice could not only positively affect the detection rate of BC while decreasing reading time, it could also allow the use of less expensive modalities without compromising diagnosis.

#### 2.3.3. AI in Breast Cancer Risk Prediction

Being able to predict the risk of developing BC would be an important step to diminishing the nefarious effects of this disease, along with its mortality rate. Several works aimed to do that, and some of them are deeply described in [36]. Here, a brief summary of several applications of ML and Deep Learning (DL) is carried out.

The concept of risk is most of the time associated with genetic or environmental risk factors. It was with that state of mind that a group of researchers [8] aimed to differentiate mammograms into high-risk (15 instances) and low-risk (143 instances) women, based on their BRCA1/BRCA2 mutation status. As it can be perceived, women with a mutation in these genes were associated with the high-risk group, while the low-risk group was composed of women that had a lifetime risk (through Gail’s model) lower than 10% and had no family history. The differentiation between these two groups was performed in a fashion similar to what was observed for lesion classification. First, there is a manual ROI definition—here, once no lesion exists, usually the area immediately before the nipple is chosen—then, feature extraction can be performed. Not only GLCM but also features directly related to pixel intensity were retrieved from the defined ROI. The feature selection procedure resulted in four significant features—two based on intensity and two GLCM. These features were used in a Linear Discriminant Function approach, which yielded an AUC of 0.91. Despite the limited size of the dataset, the authors point out that ROI size is one of the main limitations. In addition to that, the simple definition of an ROI is itself a major limitation. Breast parenchyma is a heterogeneous tissue, and analyzing just a specific region does not take into account the complexity of the parenchyma, hence limiting the obtained results and the conclusions that can be drawn.

After several years, with approaches similar to what was seen, the idea of using just a single ROI was outdated. Apart from that, instead of looking for differentiation into two risk groups, the ambition of predicting a near-term risk of developing BC started to be pursued. Tan et al. [37] evaluated the probability of a woman developing BC through the analysis of a negative mammogram. In order to do that, they gathered sequential mammograms for a group of women and gave a label to the “prior” mammogram based on the “current” mammogram. All the “prior” mammograms were negative, so the women were divided into different groups according to their assessment in the most recent evaluation. This evaluation could be: “positive” (283 women), “recalled but later proved benign” (349 women), and “negative” (362 women). After creating the dataset, feature extraction could be performed, and it was carried out not only across the entire breast but also from segmented dense regions (zones in breast tissue that encompass pixel values above the median of the entire breast). The extracted features were not simply intensity-based and GLCM but also RLM features. Moreover, the authors extracted “histogram cumulative projection” features, which are briefly described in said paper. Contrary to what was conducted in previous research, features were retrieved not just from the breast which is going to be classified but also from the contra-lateral breast. Then, using the corresponding features from each breast, asymmetry features were calculated through Equations (1)–(3).

(1) $$F_{Asymmetry1-60}=\frac{|f_{i}-g_{i}|}{max(f_{i},g_{i})}$$

(2) $$F_{Asymmetry61-120}=\left|f_{i}-g_{i}\right|$$

(3) $$F_{Asymmetry121-180}={\left|f_{i}-g_{i}\right|}^{3}$$

In order to train the classifier, these 180 features plus three risk factors (age, family history, and breast density) were passed through a feature selection routine. After that—10 features were chosen as relevant across different groups—an SVM classifier, validated through 10-fold cross-validation with a Gaussian kernel was used [38]. When aiming to differentiate the three subgroups, the classifier achieved an AUC of 0.725, which dropped to 0.716 when considering only the first and third subgroups. These results are very promising if it is kept in mind that the images used for classification do not have any lesions. Once some limitations are overcome, it could be possible to achieve more robust results: the use of real-world data instead of a laboratory-curated set, the use of different data to select features and to evaluate performance in order to avoid bias, and the use of features that not only represent asymmetry but also the effective texture of the breast.

The idea of looking for different areas across the breast was further developed by another research [39] that uses a lattice-based approach. A grid of structural elements is displayed superimposed on the breast, and texture features are extracted from each of those elements, hence describing the entire parenchyma heterogeneity. The study used 106 cases (contra-lateral healthy breast images from women with cancer) and 318 controls. The differentiation between these two groups was aimed at using a logistic regression model. The authors obtained an AUC value of 0.85 for this differentiation which outperformed the single-ROI approach. After analyzing the impact of previous mammograms for risk prediction, the positive results obtained with this research give insights into the use of contra-lateral unaffected breasts to assess the risk of future development of BC.

The use of contra-lateral continued to be used and motivated Tan et al. [40] to verify if this approach also provided positive results for Asian populations. The methodology is very similar to what was already described for past works. It should be noted that in addition to the commonly extracted texture descriptors, new features are introduced and described in this work. Texture features alongside %PMD were used to train a Linear Discriminant Analysis classifier. The obtained AUC was slightly lower than for previous studies (0.68) but gave confidence that this kind of approaches can be translated to different ethnicities.

With the advance of technology, Deep Learning can play a major role in risk prediction. With that in mind, Qiu et al. [41] aimed to predict, based on a “prior” mammogram, the likelihood of a woman being evaluated as positive in a “current” mammogram. In order to create the dataset, sequential images for the same women were needed. All the “prior” images were evaluated as negative and the division into different groups was made based on the evaluation performed on the “current” mammogram (normal or cancer). In order to achieve the purpose of the work, the authors propose a Convolutional Neural Network (CNN). The CNN has 8 layers and aims for both feature learning and classification. When it comes to feature learning, three convolutional–pooling pairs are used. The result of passing through these pairs is a feature map of dimensions 5 × 5 × 6, which is directly fed to the classifier—a multiple layer perceptron that was optimized with mini-batch statistic gradient descent. It is this classifier that will generate a score of how likely it is that the analyzed mammogram will develop cancer until the following assessment. In terms of results, this approach had a sensitivity of 0.703 and a specificity of 0.60. The overall accuracy was 71.4%, while the AUC value was 0.697. This approach, as others with DL, allows overcoming problems with the laborious work of defining, computing, and choosing handcrafted features. Of course, this work has some limitations, with the most evident being the fact that the CNN only has 8 layers, being relatively shallow. The use of a deeper network could allow learning more abstract features, which could better describe and differentiate the defined groups.

A more recent study by Yala et al. [42] aimed to develop a DL model that used both mammograms and risk factors (breast density and patient age) to predict the risk of developing BC. In order to do this, they used 71,689 images for training (2732 positive vs. 68,957 negative), 8554 for validation (316 positive vs. 8238 negative), and 8869 for testing (269 positive vs. 8282). Actually, the authors developed three different models. One was a logistic regression containing only the risk factors (RF-LR), an other was an image-only DL model—using a ResNet18 architecture—that predicted breast cancer within 5 years. Finally, the last model was a hybrid DL model that combines information from the previous two models. The RF-LR model had an AUC of 0.67, while the model that used only the mammograms had an AUC of 0.68. When combining this information, the hybrid model achieved an AUC of 0.70. Nonetheless, all of the models outperform the Tyrer–Cruz risk model, commonly used in clinical practice.

The higher complexity of risk assessment in comparison with lesion detection explains the lower results obtained in this section. However, the obtained results give confidence in pursuing these types of approaches, which can be extremely important in personalizing screening routines, potentially decreasing treatment aggressiveness and costs, and ultimately decreasing BC mortality.

## 3. AI and Data Augmentation

Learning-based systems usually train on large amounts of data to learn every crucial aspect of the input images and output accurate and robust responses. However, most entities do not have access to these large and high-quality datasets, either because they are not publicly available or they are not easy to collect. However, even those that have such data have extreme difficulties manually labeling it all.

Regarding this necessity, there is a sector dedicated to this purpose where works use generative adversarial networks (GANs) to generate synthetic samples to create high-quality datasets, in this case, mammograms. These generated mammograms have to at least match a similar texture, aspect, and styling when compared with real mammograms.

**Figure 3:**
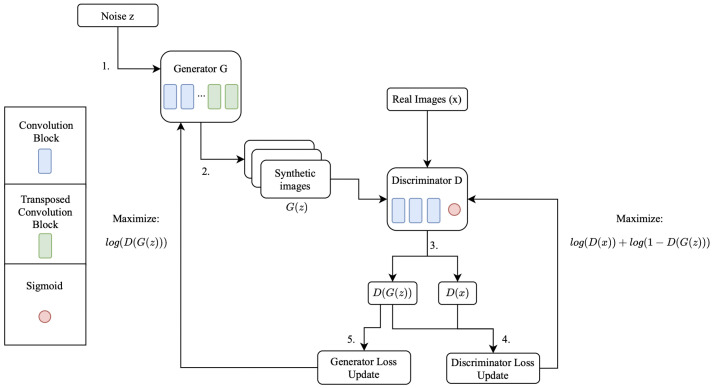
Outline of a GAN algorithm.

Following the explanation of Kunfeng Wang et al. [43], and the base work proposed by Ian Goodfellow [44], as well as looking at the schema presented in Figure 3, GANs-outline given in Figure 3 are essentially a zero-sum game between two neural networks, the generator and the discriminator, where the goal is to estimate the potential distribution of real images and generate new samples from that distribution. Despite the different flavors of GANs, all of them follow the core working idea. The generator iteratively generates images from a vector noise z, those images being referenced as G(z) on the presented schema. In each of those iterations, the discriminator receives the original images x, outputting the probability of the original images belonging to the original dataset (referenced as D(x)) and the probability of the generated images belonging to the original dataset (referenced as D(G(z))). These probability values are used to update the networks accordingly as it is possible to see in the schema, where the objective of the discriminator is to maximize the prediction of real images being predicted as real (log(D(x))) and maximize the probability of fake images being predicted as fake (log(1−D(G(z)))). As for the generator, the objective is to maximize the probability of its generated images being predicted as real by the discriminator (log(D(G(z)))). This process goes on until convergence is achieved, that is, the point where the probability of the discriminator predicting something correctly is fifty percent.

The review made by Richard Osuala et al. [45] is extraordinary since it presents many successful cases of different GAN flavors, challenges, and future improvements, as well as how GANs can be used in cancer imaging. Although it is not focused specifically on breast cancer, the 163 analyzed papers can be extrapolated and adapted to mammograms.

In addition to presenting different GAN variations for different purposes, it also states the biggest challenges in generative tasks for medical imaging, these being: the small size and complexity of cancerous lesions that may impact the quality of the generation; the high heterogeneity between tumors as well as between patients and cancer types; annotating, delineating and labeling cancer images at a large scale; inputs with high imbalanced data; and gathering large consented datasets from highly vulnerable patients.

Additionally, there are works focused on breast cancer that show the power of GANs. For these, it is important to understand how they use GANs to solve a specific problem, and how each approach can help create better datasets for specific tasks towards a future with more AI-integrated services.

Dimitrios Korkinof et al. [46,47] published a paper that uses a progressive GAN (PGGAN) for mammograms synthesis, achieving high resolutions of up to 1280 × 1024 pixels and achieving good results in five statistical moments (mean, variance, skewness, kurtosis, and hyperskewness) when assessing the similarity between the low-level pixel distributions of real and synthetic images. In addition to that, they also conducted a user study to validate if the generated images could be distinguished from the original ones, in which they observed a binomial distribution with success probability π = 0.5 (*p* = 0.999, Chi-square test), indicating that participants were unable to distinguish between generated and real mammograms. The core idea behind this variant is essentially making the generator grow throughout the training, outputting images at different scales until it reaches the desired output scale. They used proprietary images that were down-sampled by the largest factor to match one of the desired dimensions and padded the other dimension with zeros. According to the authors, even though the results were good, training such networks is difficult since it requires achieving the Nash equilibrium [48]. Not just that, but most times the training process can be unstable and susceptible to mode collapse and gradient saturations.

The work of Rui Man et al. [49] also focuses on generating synthetic samples, but in this case, they generate patches of a histopathological image. This work, mostly called AnoGAN (Anomaly Detection GAN), brings many advantages to training classification systems for cancer imaging. They show that adding such generated samples to a classifier dataset considerably increases metrics such as accuracy, precision, recall, and f1. The dataset used here was the BreaKHis dataset, where they performed the stain normalization process and then performed patch extraction and standard data augmentation. Some values of these metrics, considering the use of Densenet121 [50] classifiers with and without the generated samples from AnoGAN at different magnification levels, were the following: for a magnification of forty times (40×), the values of accuracy were 94.26 ± 3.2% without using the samples from AnoGAN and 99.13 ± 0.2% with the generated samples; for a magnification of one hundred times (100×), the values of accuracy were 92.71 ± 0.4% without using the samples from AnoGAN and 96.39 ± 0.7% with the generated samples; and for higher magnifications and so on, the values follow the same idea of better results with the addition of the generated images.

Contrary to these two works, a different research conducted by Xiangyuan Ma et al. [51] focuses on generating samples of mammogram lesion segmentation masks. This allows combating one of the biggest challenges when constructing a dataset, which is image labeling. Generating segmentation masks for the respective input image facilitates this process, and in the medical area, this is extremely important since it does not require huge amounts of time spent by doctors to identify and segment lesions. For this work, they collected samples from a health entity and then pre-processed them by scaling the gray-level dynamic range and resizing them to 256 × 256 pixels. They used the Sørensen–Dice coefficient (DSC) and the Jaccard Index (JI) to evaluate the results and showed how much better generating the segmentation masks with GANs surpassed well-known networks such as U-Nets [52], where on average the DSC and JI values for this approach achieved 87.0 ± 7.0% and 77.6 ± 10.1%, respectively, whereas a Baseline U-Net model just achieved DSC and JI values of 81.1 ± 8.7% and 69.0 ± 11.3%, and an Improved U-Net model just achieved DSC and JI values of 85.7 ± 8.6% and 75.9 ± 11.8%. In addition to this, they also evaluated the impact of such results on the background parenchymal enhancement.

In light of these works, there are other works such as the one conducted by Eric Wu et al. [53] that focus on obtaining more image variation. Usually, a regular GAN implementation may not give enough variation to what is required to create a robust computer-aided diagnosis system. Therefore, the samples generated in this work are manipulated to either add malign tissues to the resulting image or to remove them. This greatly boosts variability and increases the performance of computer-aided diagnosis systems. They used the DDSM (Digital Database for Screening Mammography) dataset, and reduced images to a resolution of 1375 × 750 pixels. Similarly to the metrics used to evaluate the results from [49], they used a classification network to assert the predictive quality by using datasets with and without these new variations, the scarcity of data being their major limiting factor. In terms of accuracy values, it was possible to see that the non-use of these generated samples resulted in accuracy values of 0.882 for no augmented datasets and 0.887 for traditional augmentation datasets, whereas combining traditional augmentation and these varied samples resulted in an accuracy of 0.896.

In addition to these, there are works such as the one conducted by Caglar Senaras et al. [54], whose modus operandi resembles an image-to-image translation. It allows the user to give a segmentation mask as input (a histopathological image segmentation) and make the generator output samples that match that exact mask. Even though the variability is always conditioned by the user input, this is a great way to generate more varied samples and increase the quality of a dataset. For this, they also collected a dataset from a health entity, and all of the image regions of interest were divided into tiles of size 256 × 256. To evaluate such results, the team built a hierarchical logistic regression model that indicated that the average reader would be able to correctly classify an image as fake or real more than 50% of the time with a probability of 44.7%, indicating clearly that overall professionals are not able to distinguish both; thus, these are a great addition to increase the variability of any dataset using the same image type.

Last but not least, there are many other projects, similar to the ones described above, that focus on GANs applied to breast cancer and that achieve great results. Some of them are “Breast Ultrasound Image Synthesis using Deep Convolutional Generative Adversarial Networks” [55], “A generative adversarial network for synthetization of regions of interest based on digital mammograms” [56], and “RDA-UNET-WGAN: An Accurate Breast Ultrasound Lesion Segmentation Using Wasserstein Generative Adversarial Networks” [57].

To sum up, even though the results from the papers mentioned above are remarkable, when developing a project in the breast cancer area using GANs, the necessity to validate the results is extreme. The synthetic images constantly need to be validated in the first phase with a classifier and then validated afterwards by a real doctor to ensure such images seem real and correspond to possible mammograms.

## 4. Self-Supervised Learning

Self-Supervised Learning (SSL) has been pushing the limits of unsupervised learning in many domains, from natural language processing to traditional computer vision tasks. Deep Learning usually works very well when there are large labeled datasets to train the model, which is known to be difficult to obtain in medical imaging for many pathologies. SSL attempts to diminish the need for large labeled datasets by learning rich representations only from the data itself. The main idea of SSL is to learn from a designed task that can be described as filling in the blanks, i.e., to predict any part of the input data that is hidden from any other part. The representations learned by solving this prediction task are then used to train, in a supervised manner, an additional network on a given task. If the SSL task learned good features, then the amount of labeled data used to solve the downstream task can be reduced considerably. This can be performed in several flavors, and in this section we mainly focus on its application to breast-related tasks.

Contrastive learning is one of the most popular SSL algorithms in natural language processing and computer vision. The main idea is to learn representations by contrasting positive pairs of images against negative pairs. The comparison is usually performed in the feature space. Each image is represented by a feature vector resulting from a forward pass through an embedding network, and then both vectors are compared using some metric or loss. The network leans through minimizing or maximizing the chosen metric in case of positive or negative pairs, respectively.

In Li et al. [58], the authors address the task of lesion detection using mammographies and particularly focus on augmenting the generalization capability of the network to different machine vendors. The goal is to learn features that are invariant to multiple styles and views produced by different vendors, resulting in a model that generalizes across domains. There are two main steps in this approach: the self-supervised feature learning step and the supervised fine-tuning used to learn the lesion detection task. Most of the learning should happen during the first step, which regards the multi-view and multi-style generalization. This work uses CycleGAN [59] to generate multiple images from different vendors from a single vendor. These constitute the positive pairs once they show the same view represented by different vendors. Additionally, the CC and MLO views of the same breast are also regarded as positive pairs. This method was trained using mammograms of three vendors and tested on an unseen fourth vendor, as well as in the INbreast dataset.

In Gao et al. [60], the authors propose a method to enhance the contrast of areas of potential lesions. This procedure is usually referred to as mammogram normalization into pathology-aware style. The main goal is to facilitate downstream tasks such as lesion classification and segmentation. One of the main challenges of this process is to adapt it to different types of breasts in terms of density. The method consists of an encoder with two decoder heads that produce two images: *Y*, the high contrast mammogram, and *m*, a map to be multiplied with *Y*. It is possible to reconstruct the original image *I* using *Y* and *m*. The difference between the reconstructed $\hat{I}$ and the real input image *I* is used to train the encoder–decoder network.

Miller et al. [61] addressed the problem of breast cancer detection using SSL, reaching an efficiency improvement of labeled data by nearly 4-fold, while still generalizing across datasets. This paper proposed an SSL-based pre-training to learn features to be used in patch and whole-image classification. It also benchmarks four popular SSL methods, of which SWaV, a clustering-based method, was the best. This may be due to the fact that it is harder for this method to exploit shortcuts, such as background artifacts, to solve the pretext task. This is a common problem in SSL and is highly problematic and modality-dependent. Due to the large size of mammograms, the images were split into patches to train the networks. The encoder networks were then applied to the whole image to produce a grid of feature vectors, which can be aggregated with some pooling methods. The authors also experimented with replacing global pooling with attention and self-attention mechanisms, which also improved performance, which is consistent with results in other computer vision tasks.

Ouyang et al. [62] address the task of detection of clustered microcalcifications in mammograms. The identification of these structures is crucial because they are one of the first signs of breast cancer but also very difficult due to the high variability of the clusters in size and distribution. This paper proposes a method based on the well-known U-Net architecture, trained end-to-end in a multi-task fashion to identify the microcalcification clusters and simultaneously segment and classify them. The identification of microcalcification clusters is performed using the Class Activation Mapping method, which produces an attention map derived from the image according to the malignancy score of the pixel. The localization of the clusters is refined with an attention loss. The result of this localization is then used to feed a self-adversarial learning module, which distinguishes between benign and malign regions within the same image, and improves the classification accuracy of the backbone network.

Self-supervised learning can benefit from other engineering techniques and learning methods that have been used in supervised learning. For example, in [63], Srinidhi and Martel proposed a curriculum learning approach to the self-supervised learning process by progressively sampling harder examples. There are two main questions to address in curriculum learning: how to score the example difficulty and in what order to present them to the network. The difficulty of each sample is assumed to be correlated with the loss value associated with it. The hardness score decreases with time, in what the authors call dynamic hardness-aware loss, which serves to order by difficulty and sample according to a strategy. The authors first train with easy-to-hard examples and then with hard-to-very-hard examples, ordered according to the aforementioned score. This method was tested on three histology datasets, improving the performance especially on out-of-domain distribution data.

It is standard practice to reuse features learned in a different domain or task and adapt them to the task at hand. This process is called transfer learning. Deep neural networks trained on the large ImageNet dataset are widely available, and their features are used for the most varied tasks using natural images. In [64], Truong, Mohammadi, and Lenga investigate the transferability of self-supervised features in medical image classification tasks. The authors conclude that the self-supervised pre-trained models perform better than the supervised models in tasks such as tumor detection, diabetic retinopathy classification, and multiple chest pathology classification and detection. This is particularly important for small labeled datasets.

Table 1 presents a summary of the reviewed articles.

## 5. Discussion

This paper covers several areas in breast imaging where AI can play a major role. In terms of lesion detection/interpretation, Machine Learning methodologies based both on ROI and overall breast analysis have proven to provide positive outcomes. The strategy of developing handcrafted features is not only laborious, but it can miss some undercover characteristics that could help to learn. As it was perceived, most of these ML studies had a small dataset, which makes the present metrics not robust enough for clinical practice transition.

The shift from ML to DL, with increased computational capacity, could help to overcome some problems that block the deployment of AI solutions in clinical practice. Deep Learning techniques can not just use a great amount of data (which increases the robustness of the evaluation made) but can also learn representations from raw input (even in an SSL fashion as seen in the previous section), unveiling patterns that would not be found with typical handcrafted features. Of course, there are several challenges before AI can be correctly implemented in clinical practice [65,66,67]: in addition to the need for a big dataset correctly labeled and with good image quality, the true generalization capability of the models needs to be assessed as well. This generalization capability is usually evaluated with a test set that was not used for training but that comes from the same distribution of the training set, which could bias evaluation. Therefore, novel AI solutions that aim for clinical deployment should be concerned not only with gathering a great amount of data but also with increasing their variability in terms of machinery used for acquisition, lesion type, and women’s ethnicity.

As it was discussed in this paper, data augmentation techniques could in fact help to overcome the problem of lack of data. Nonetheless, data augmentation techniques need to be further studied and developed so that they can learn to correctly reproduce the specific characteristics of the lesions that it is trying to augment. If an AI system can achieve a substantial performance and generalization capability, further testing in a real-world environment is needed in order to guarantee not only that the software is doing what it was designed to do but that it is safe. This testing period is also important for the users, since it allows them to understand what they can expect from the AI system, how it works, and even find some limitations, enabling safer use. Despite these current challenges, AI has great potential for reducing the workload of healthcare professionals, while increasing detection rates.

The work presented here is a review of different topics where AI can positively impact breast imaging. As it was discussed, it was chosen to give a general review of different topics instead of extensively describing each area of research. This could be a limitation of this study since it may overlook divergent strategies to achieve the same goal. Future work should focus on exploring each of the areas identified in the present work, constructing a repository of the most significant research in each area. Nonetheless, the paper gives a general review of the methodologies used in the development of ML/DL techniques for lesion identification/interpretation and for breast cancer risk prediction. A section on data augmentation presents several studies that can be of extreme importance in overcoming problems related to lack of data. Therefore, this review, despite gathering into one article different uses that AI can have in breast images, also serves the purpose of being a basis for novel research that aims to overcome the problems identified with each present work.

## 6. Conclusions

There are several areas in the field of breast cancer that can benefit from the use of AI. Lesion diagnosis and detection can be improved and sped up either through traditional ML/DL methodologies or through SSL. The incorporation of AI in the medical field, especially in what concerns diagnosis, can be of great importance since the timing is extremely important. The fact that these AI methodologies can serve as a “second reader” of the medical images allows reducing the time spent in assessing each image while improving accuracy. The differentiation between normal and abnormal tissue is very well-achieved through AI using mammography, as seen in [32]. In addition to that, the work conducted in [33] also shows the potential that AI has in being able to correctly characterize lesions. Given what was seen when analyzing [34,35], AI can also be applied in imaging techniques other than mammography while maintaining positive results. Of course, the translation of these latter methodologies to clinical practice is more difficult since the used imaging modalities are not standard.

There are several risk prediction models used in clinical practice, most of them concerning genetic factors. The capability of developing an AI system that can predict the future development of BC would be of extreme importance once it could allow for adapting screening modalities, detecting the disease earlier, resulting in an improved prognosis. The results obtained through ML techniques in the analyzed papers [8,37,39,40] give confidence that these types of systems can be developed, either referring to genetic information, contra-lateral unaffected images, or sequential mammograms. However, the laborious pre-processing and the need for image segmentation might be barriers to their implementation in clinical practice. On the other hand, DL methodologies, such as the ones seen in [41,42], overcome some of the referred problems while presenting satisfactory results (still with room for improvement).

Nonetheless, in order to develop these DL techniques, big databases of annotated data are needed. Most of the time, it might be difficult to find these databases, and that is why the works reviewed in Section 3 and Section 4 are so important. The great range of applicability that GANs have in the field of data augmentation should be noted. In addition to the generation of synthetic mammograms that resemble real images—so much so that readers cannot differentiate them—these techniques can also be applied to histopathological and MRI images, always with good results.

GANs are not only highly useful in the augmentation of the amount of data but also in their variability, since they have the capability to generate new images with malignant lesions added or to remove the lesions from images that already have them. Self-Supervised Learning, on the other hand, tries to overcome the need for labeled data by learning characteristics of the unlabeled input data, and then those learned representations can be used to train models in a supervised fashion. The assessed works have a wide range of applications, which really translate the potential that AI can have in the medical field—the capacity to, from an image of one vendor, create multiple images from different vendors; enhance contrast in areas of the images that have a greater potential for the appearance of lesions; and, of course, breast cancer detection improvement.

As it can be perceived, AI can be used in different fields concerning breast imaging and most of the time with very encouraging results. The transfer of these AI solutions to day-to-day clinical practice could result in better outcomes for the patients while diminishing the workload of healthcare professionals. The limitations of the analyzed studies open the door for further investigation and future work.

Since the current medical paradigm is one of preventive and personalized care, the development of methodologies that in an individual way predict the risk of developing BC should be of great importance. However, solutions such as this one should overcome many of the flaws presented by the reviewed works. The datasets used should be as diverse as possible both in terms of breast anatomy and machinery used. In addition to diversity, increasing dataset size is also of extreme importance in order to achieve results that can be translated to clinical practice. The transition from handcrafted features that could bias learning to self-learned representations as it happens with Deep Learning should also be considered, while using entire images for classification, leaving behind the laborious ROI definition. These future lines of work, if correctly employed in clinical practice, can have an extremely positive impact on the lives of thousands of women.

## Figures and Tables

**Table 1:** Studies Summary.

Authors	Goal	Method/Algorithm	Imaging Modality	Results
Kayode et al. [32]	Benign/Malignant Lesion Differentiation	Texture Features with SVM	Mammography	Sensitivity = 94.47%; Specificity = 91.3%
Mohanty et al. [33]	Benign/Malignant Lesion Differentiation	Texture Features with Decistion Tree	Mammography	AUC = 0.995
Wei et al. [34]	Benign/Malignant Lesion Differentiation	Texture Features/LBP with SVM	Ultrasound	Sensitivity = 87.04%; Specificity = 87.62%; AUC = 0.9342
Nie et al. [35]	Benign/Malignant Lesion Differentiation	Texture/Morphology Features with Artificial Neural Network	MRI	AUC = 0.82
Huo et al. [8]	High-Risk/Low-Risk group Differentiation	Texture Features with Linear Discriminant Analysis	Mammography	AUC = 0.91
Tan et al. [37]	Risk Prediction based on a “prior” evaluation	Asymmetry Texture Features/risk-factors with SVM	Mammography	AUC = 0.725
Zheng et al. [39]	Differentiate contra-lateral healthy images from diseased women from normal cases	Texture Features with Logistic Regression	Mammography	AUC = 0.85
Qiu et al. [41]	Risk Prediction based on a “prior” evaluation	CNN	Mammography	Sensitivity = 70.3%; Specificity = 60%; AUC = 0.697
Yala et al. [42]	Single-Image + Risk Factors Risk Prediction	CNN (ResNet18)	Mammography	AUC = 0.7
Dimitrios Korkinof et al. [46,47]	Mammogram Synthesis	PGGAN	Mammography	≈50% probability of identifying synthetic samples
Rui Man et al. [49]	Mammogram Patches Synthesis	AnoGAN	Histopathological	Classifiers with >99% accuracy
Xiangyuan Ma et al. [51]	Segmentation Masks Synthesis	GAN	Mammography Segmentation Masks	Dice-Coefficient > 87%; Jaccard Index > 77%
Eric Wu et al. [53]	Mammogram Variation	GAN	Mammography	Classifiers with accuracy of 89.6%
Caglar Senaras et al. [54]	Image-to-Image Mammogram Synthesis	GAN	Mammography	≈50% probability of identifying synthetic samples
Li et al. [58]	Lesion Detection	SSL, GAN and CNN	Mammography	Improvements of ≈3 pp on accuracy
Gao et al. [60]	Normalization, classification and segmentation	SSL and CNN	Mammography	Improvements of ≈10 to 15 pp on AUC scores
Miller et al. [61]	Breast cancer detection	SSL and CNN	Mammography	Improved 4-fold data efficiency and ≈3 pp on accuracy
Ouyang et al. [62]	Detection of clustered microcalcifications	SLL and CNN	Mammography	Improvements of ≈5 pp on AUC scores
Srinidhi and Martel [63]	Classification	SSL, curriculum learning, CNN	Histology	Improvements of ≈2 pp on AUC scores
Truong et al. [64]	Classification and Detection	SSL and CNN	lymph node images, fundus images, and chest X-ray images	Improvements of ≈2 pp on AUC scores

## Data Availability

Not applicable.
